# Withanolides Are Detected in Human Urine Following Oral Administration of a *Withania somnifera* Product

**DOI:** 10.3390/ijms27125289

**Published:** 2026-06-11

**Authors:** Alex B. Speers, Ellala D. Limoico, Axel Lozano-Ortiz, Luke Marney, Jaewoo Choi, Sarah A. Barr, R. Thomas Williamson, Wendy K. Strangman, Claudia S. Maier, Amala Soumyanath

**Affiliations:** 1 BENFRA (Botanicals Enhancing Neurological and Functional Resilience in Aging) Botanical Dietary Supplements Research Center, Oregon Health & Science University (OHSU), Portland, OR 97239, USA; 2 Department of Neurology, Oregon Health & Science University (OHSU), Portland, OR 97239, USA; 3 College of Liberal Arts and Sciences, Portland State University (PSU), Portland, OR 97201, USA; 4 Department of Biology, Portland State University (PSU), Portland, OR 97201, USA; 5 Department of Chemistry, Oregon State University, Corvallis, OR 97331, USA; 6 Department of Chemistry and Biochemistry, University of North Carolina Wilmington, Wilmington, NC 28403, USA; 7 Linus Pauling Institute, Oregon State University, Corvallis, OR 97331, USA

**Keywords:** *Withania somnifera*, ashwagandha, urine, withanolides, withaferin A, pharmacokinetics, liquid chromatography, mass spectrometry

## Abstract

Ashwagandha (*Withania somnifera* (L.) Dunal; WS) is an adaptogenic herb, widely used in botanical dietary supplements. Plasma pharmacokinetics of selected withanolides, an important group of bioactive phytochemicals found in WS, have been described but no previous reports were found on their urinary excretion following WS ingestion. Healthy, older adults were administered oral doses of 240 or 480 mg of Shoden^®^, a commercial extract of WS roots and leaves, and urine was collected over the subsequent 12 h. Using validated methods employing liquid chromatography coupled to multiple reaction monitoring mass spectrometry (LC-MRM-MS), we analyzed Shoden^®^ and participant urine samples for the presence of 11 withanolides for which standards were available. Only withaferin A (0.09 µmol/mg Shoden^®^), withanoside IV (0.03 µmol/mg Shoden^®^), and a mixture of 2,3-didehydrosomnifericin and 3*R*-viscosalactone B (0.19 µmol/mg Shoden^®^) were present in Shoden^®^ powder in amounts greater than 0.01 µmol/mg Shoden^®^. We detected four withanolides in participant urine: withaferin A; sominone, the aglycone of withanoside IV; 3*R*-viscosalactone B; and an abundant unidentified metabolite (WFNX), isomeric with 4-oxo withaferin A, and not detected in Shoden^®^. Urinary recovery of the other 3 detected compounds was generally low (<3% of the amount in Shoden^®^). These results provide new insights into the oral absorption, metabolism, and urinary excretion of withanolides found in WS and highlight additional withanolides to consider in future human pharmacokinetic and bioactivity studies of WS products.

## 1. Introduction

Ashwagandha (*Withania somnifera* (L.) Dunal; WS), an adaptogen commonly used for stress-related disorders [1], is one of the best-selling botanical dietary supplements in the United States [2]. The biological effects of WS have largely been attributed to withanolides, a group of steroidal lactones that are abundant in WS and whose oral absorption has been demonstrated in animals and humans [3]. Human pharmacokinetic studies have detected the withanolides withaferin A [4,5,6,7], withanolide A [4,5,6,7,8], withanoside IV [4,5,6,7], withanoside V [6], sominone [6], and 12-deoxywithastramonolide [6,7,8] in the plasma following oral administration of various WS compounds. Ramapalaniappan et al. reported total withanolides (the combined plasma concentrations of withaferin A, withanolide A, withanoside IV, and 12-deoxywithastramonolide) but did not report individual concentrations for those four withanolides [9]. While these human studies have provided critical data related to the oral absorption of WS, none of these studies have measured the urinary excretion of withanolides, data which are necessary to fully understand the oral absorption, metabolism, and excretion of withanolides from WS in the human body. To our knowledge, the only human-level evidence for the urinary excretion of withanolides comes from a pharmacokinetic study of *Physalis peruviana* (golden berry), in which the withanolide 4β-hydroxywithanolide E was detected in participant urine collected over 24 h following oral administration of 250 g of golden berries [10].

**Figure 1:**
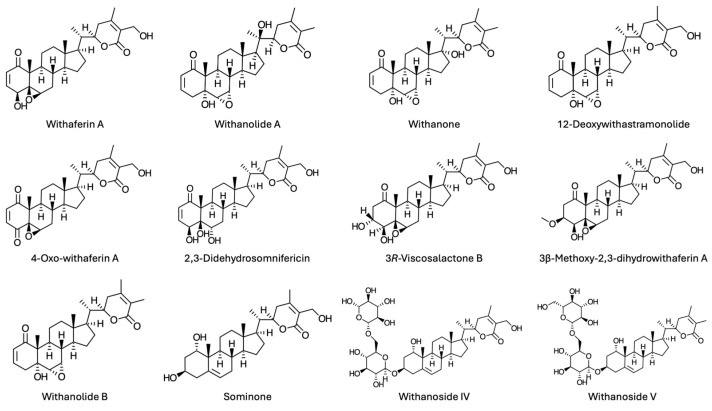
Commercially available withanolides selected as standards for analysis, and 3*R*-viscosalactone B.

In previous studies on the plasma pharmacokinetics of withanolides, liquid chromatography coupled to multiple reaction monitoring mass spectrometry (LC-MRM-MS) has been the most widely used method [3]. Here, we report on the development and validation of an LC-MRM-MS method for the analysis of 11 withanolides (Figure 1) in human urine and its application to urine samples collected over 12 h during a pharmacokinetic study of Shoden^®^, a commercially available extract of WS root and leaves.

## 2. Results

### 2.1. Method Development and Validation

An LC-MRM-MS method was developed to separate 11 withanolides using a 20-min reversed phase chromatographic system coupled to their detection using characteristic mass spectral fragmentation patterns. The retention times (Rt) and optimum transitions of these 11 compounds are shown in Table 1. Four isobaric withanolides were well separated in this system: withaferin A (Rt: 7.41 min), 12-deoxywithastramonolide (Rt: 7.96 min), withanolide A (Rt: 8.12 min), and withanone (Rt: 8.29 min), allowing them to be differentiated even though they were sometimes detected with the optimum MS/MS transitions of another withanolide.

For each withanolide, the lower limit of quantitation (LLOQ), upper limit of quantitation (ULOQ) and linearity of detector response (area ratio to internal standard) as a function of urinary concentration between the LLOQ and ULOQ is shown in Table 1. The equations for the calibration curves are given in Supplementary Table S1. LLOQ and ULOQ were determined based on FDA guidelines for precision and accuracy [11] as well as observed deviation from linearity as described under Methods (Section 4.3). Interday variability was investigated for compounds of particular interest in this study (withaferin A, sominone, 4-oxo withaferin A and 2,3-didehydrosomnifericin) by comparing slopes of the calibration curves constructed using standards on two separate days. A percentage difference of 40% to 160%, depending on the analyte, was observed between the slopes of calibration curves constructed on two different days (Supplementary Table S1), indicating interday variability in instrument sensitivity. Therefore, a practice was adopted of running calibration standards alongside analytical samples for every separate run. Chromatograms demonstrating the selectivity of the method for the four withanolides (or their isomers) that were detected in participant urine are shown in Supplemental Figure S1.

### 2.2. Study Participants

Four participants (Table 2) completed at least one pharmacokinetics visit and provided urine collected over 12 h post-Shoden^®^ (240 mg or 480 mg) administration. All four participants (1 male, 3 female) were non-Hispanic White, had an average age of 71.3 years (SD 4.3 years), and an average body mass index of 24.2 (SD 3.4). Only one of these participants (Male A) completed separate pharmacokinetics visits for each dose. For two participants, the second pharmacokinetics visit was halted early due to mild anemia on the baseline labs (*n* = 1; Female B) and intervention-related diarrhea and vomiting at the 480 mg dose (*n* = 1; Female A). The second pharmacokinetics visit was not scheduled for Female C because the participant withdrew from the study due to intervention-related diarrhea and vomiting at the 480 mg dose. Budget and time constraints prevented the enrollment of additional participants.

### 2.3. Four Withanolides Detected in Urine

Of the 11 withanolides that were analyzed, withanolide B, withanolide A, withanone, 12-deoxywithastramonolide, 3β-methoxy-2,3-dihydrowithaferin A, withanoside IV, and withanoside V were not detected in any participant urine sample, at either dose level of Shoden^®^. Four compounds were detected: withaferin A, sominone, and two other compounds that were detected using transitions corresponding to 2,3-didehydrosomnifericin and 4-oxo withaferin A, respectively. However, both latter compounds detected in urine had slightly shorter Rt values than the standard compounds (Supplementary Figure S1). The Rt values were 6.39 min and 6.15 min, respectively, for 2,3-didehydrosomnifericin and the urinary compound, and 9.59 min and 9.30 min, respectively, for the 4-oxo withaferin A standard and the related compound detected in urine (Supplementary Figure S1). Further exploration using LC-HRMS (Supplementary Figure S2) demonstrated that these unknown withanolides, as well as the corresponding purchased standard compounds, had high-resolution masses within ±1 ppm of the predicted mass for the standards (489.28468 for 2,3-didehydrosomnifericin, and 469.25647 for 4-oxo withaferin A). This value was well within the ±5 ppm limit required by the American Chemical Society [12] to confirm molecular formula and established that the two unknown urinary compounds were structural isomers of the corresponding standards.

The compound isomeric with 2,3-didehydrosomnifericin was compared by LC-MRM-MS with the hydrolysis products of withaferin A (Supplementary Figure S3) and was found to correspond to 3*R*-viscosalactone B, which had previously been identified using ROESY NMR [13]. This compound was quantified in participant urine as 2,3-didehydrosomnifericin.

The compound isomeric with 4-oxo withaferin A (WNFX) was quantified as 4-oxo withaferin A but was not identified. A comparison of the fragmentation patterns of WFNX and 4-oxo withaferin A using LC-MS/EPI MS (enhanced product ion mass spectrometry; Supplementary Figure S4) showed several fragments common to both compounds (m/z 451, 433, 297, 279, 123, and 67), which varied in their relative abundance. While the base peak was the molecular ion (m/z 469) for both compounds, the most abundant fragment was at m/z 123 for 4-oxo withaferin A and at m/z 297 for WFNX. Based on a detailed interpretation of the mass spectrum of withaferin A [14], fragments at m/z 451 and 433 correspond to the loss of one or two molecules of water respectively, while the m/z 297 fragment corresponds to the loss of the lactone ring by fission of the C-17/C-20 bond. The m/z 67 fragment arises from breakdown of a charged lactone moiety. The presence of these common fragments suggests that 4-oxo withaferin A and WFNX share a similar lactone ring structure. A fragment at m/z 122 reported for withaferin A using negative ionization [15] may correspond to the m/z 123 fragment seen for 4-oxo withaferin A and WFNX, but it was not identified. Differences in the fragmentation pathways of 4-oxo withaferin A and WFNX were observed in the region below m/z 279, but the corresponding moieties could not be identified. We were therefore unable to determine a structure for WFNX based on the comparative mass spectra.

Results from the quantitative analysis of participant urine samples are presented in Table 3. For each withanolide, amounts excreted (µmol) are presented with and without enzyme incubation (see Methods, Section 4.7). Results are presented for a specific analyte and internal standard transition selected based on optimum validation data (Table 3). For the quantitation of 3*R*-viscosalactone B, the participants’ urine samples were diluted with blank commercial human urine prior to analysis due to the high concentration observed in initial analyses.

The measured amounts of each withanolide in Shoden^®^ powder and the predicted amount delivered by 240 mg and 480 mg capsules are given in Table 4. While 4-oxo withaferin A was detected in small amounts at the correct retention time, detection using the transition for 2,3-didehydrosomnifericin resulted in a composite peak (Supplementary Figure S5). Separation using the same LC-MRM-MS system as used for the urine samples indicated that the composite peak was a mixture of 3*R*-viscosalactone B and 2,3-didehydrosomnifericin (Supplementary Figure S3).

## 3. Discussion

### 3.1. Urinary Excretion of Withanolides

In this study, we detected four withanolides in the 12-h urine samples of healthy, older adults administered 240 and 480 mg of Shoden^®^, an extract of WS root and leaf. To our knowledge, these are the first data confirming the urinary excretion of withanolides from WS in humans. Based on LC-MRM-MS and/or LC-HRMS comparison with standards, three withanolides were identified as withaferin A, sominone and 3*R*-viscosalactone B, while the fourth withanolide (WFNX) appears to be a structural isomer of 4-oxo withaferin A. 3*R*-Viscosalactone B and WFNX were quantified using calibration curves prepared for their isomers, 2,3-didehydrosomnifericin and 4-oxo withaferin A, respectively; therefore, their urinary excretion amounts discussed below may vary from true values.

Among the four withanolides detected, the highest amounts excreted were observed for 3*R*-viscosalactone B, with amounts ranging from 0.34–0.73 µmol (165–358 µg) at the 240 mg dose of Shoden^®^ and from 1.11–1.90 µmol (543–931 µg) at the 480 mg dose of Shoden^®^. Incubation of the urine samples with a glucuronidase/sulfatase enzyme mixture did not consistently alter amounts excreted for three of the withanolides (withaferin A, 3*R*-viscosalactone B, and WFNX; Table 3); however, sominone, the aglycone of withanoside IV, was not detectable in the urine without enzyme incubation, demonstrating that this compound is predominantly excreted as a Phase II conjugate (Table 3).

The main compounds found in Shoden^®^ powder (Table 4) were a mixture of 2,3-didehydrosomnifericin and 3*R*-viscosalactone B (93.19 ± 3.26 µg/mg Shoden^®^) and withaferin A (43.62 ± 4.38 µ/mg Shoden^®^). A low percentage of these administered withanolides were excreted in urine (0.83–2.08% for 2,3-didehydrosomnifericin/3*R*-viscosalactone B and 0.16–1.84% for withaferin A). Reasons for this could include their poor oral absorption, extensive gut or systemic biotransformation, significant biliary/fecal elimination, or their distribution and accumulation in tissues. Analysis of plasma levels following Shoden^®^ administration, which would be informative in evaluating absorption, has recently been completed and will be reported separately.

Withanoside IV and withanoside V were not detected in urine despite being the third and fourth most abundant compounds in Shoden^®^ (Table 4). For withanoside IV, this may be explained by the presence of sominone in the urine, the aglycone of withanoside IV. The percentage of administered sominone excreted in urine (19–58%) was much higher than for withaferin A and 3*R*-viscosalactone B, which could be a result of direct absorption since sominone was present in Shoden^®^ powder, but is also likely related to the hydrolysis of withanoside IV present in Shoden^®^. The amount of sominone excreted corresponds to 0.83–2.49% of the amount of withanoside IV administered in Shoden®. The aglycone of withanoside V may also have been present in urine, but the LC-MRM-MS method did not include transitions to detect this compound. WFNX was not seen in Shoden^®^ powder, suggesting that it may be a metabolite of withaferin A or another component of Shoden^®^. It is not surprising that 12-deoxywithastramonolide, withanolide A, withanolide B, withanone, and 4-oxo withaferin A were not detected in the urine given that they were found in very low amounts in Shoden^®^ powder (Table 4).

For the participant (Male A) that completed both pharmacokinetics visits (i.e., 240 and 480 mg Shoden^®^), the urinary excretion of all the detected withanolides increased from the 240 to 480 mg dose, but inconsistently. While the percentage of administered withanolide excreted in the urine more than doubled for 3*R*-viscosalactone B between the 240 and 480 mg dose for Male A (0.83% and 1.90%, respectively), increases were more modest for withaferin A (0.49% and 0.74%, respectively) and sominone (29% and 38%, respectively). The amount of WFNX excreted was higher (but not double) at the 480 mg dose (0.41 µmol) compared to the 240 mg dose (0.27 µmol). Urinary excretion of the withanolides over 12 h was not consistent between participants at the same dose level, with considerable variation noted, especially for withaferin A. For example, at the 480 mg dose, the amount of withaferin A excreted ranged from 0.05–0.79 µmol (22–308 µg) for the three participants. When considering the micromolar amounts of withaferin A and the two possible metabolites of withaferin A (WFNX and 3*R*-viscosalactone B) excreted, total urine excretion of withaferin A and its metabolites were similar between the three participants (Male A = 2.41 µmol, Female B = 2.98 µmol, Female C = 2.38 µmol). This suggests that the variability in the amounts excreted among the participants for the individual compounds may be due to differences in metabolism rather than absorption. Conversely, there was a considerable difference in total urine excretion for withaferin A and its possible metabolites for the two participants at the 240 mg dose (Male A = 0.76 µmol, Female A = 1.37 µmol), who had similar BMI values (24.43 and 22.87, respectively). Differences between individuals in the relative proportions of withaferin A and its putative metabolites WFNX and 3*R*-viscosalactone B could arise due to sex and BMI, or due to differences in gut microbiota which could contribute to the biotransformation of withaferin A. Dai et al. (2019) detected 7 metabolites with LC-MRM-MS following incubation of withaferin A with human liver microsomes in vitro arising from oxygenation, hydrolysis, or both [16]. One of three metabolites detected at parent m/z 489 may correspond to 3*R*-viscosalactone B seen in the present study. None of the seven metabolites had the same m/z value as WFNX (469). The remaining in vitro metabolites had parent m/z values of 487 or 503. Transitions with parent ion m/z 487 were not included in the LC-MRM-MS method of the current study, and the 3β-methoxy-2,3-dihydrowithaferin A transitions included here (503.3/67.0; 503.3/95) differed from those used in the the Dai study (503/297; 503/315). These in vitro metabolites would therefore not have been detected even if present in participant urine.

### 3.2. Bioactivity of the Excreted Withanolides

While the identity and therapeutic potential of WFNX is unknown, it is notable that two withanolides detected in urine (withaferin A and sominone) and the compound viscosalactone B (the 3*S* epimer of 3*R*-viscosalactone B) have all demonstrated intriguing biological activities in pre-clinical studies. Withaferin A, the first withanolide identified and isolated from WS [17], has been extensively studied for its anticancer activity, which has been demonstrated against a variety of cancer types in cellular and animal models [18]. Withaferin A has also demonstrated anti-diabetic, neuroprotective, cardioprotective, anti-hepatitis, and anti-osteoporotic effects in various pre-clinical models [19]. However, the poor bioavailability of withaferin A (due either to low absorption or extensive biotransformation) implied in the present study calls into question the translatability of the preclinical studies or could also imply that the metabolites of withaferin A contribute to its biological activity.

Viscosalactone B, a compound related to withaferin A, has similarly demonstrated anti-cancer effects [20,21,22,23,24], in addition to neuroprotective [25] and anti-inflammatory effects [26]. Sominone, the aglycone of withanoside IV, has demonstrated neuroprotective effects in cellular and animal models of Alzheimer’s disease [27,28,29]; further, in a small clinical trial (*n* = 40), a WS extract standardized to sominone content improved cognitive function in a population with mild cognitive impairment [30], suggesting sominone may modulate the cognitive effects of WS.

### 3.3. Strengths and Limitations

A strength of the present study was the inclusion of 11 withanolides in our LC-MRM-MS analysis, whereas previous human studies focused on a more limited number of withanolides (e.g., withaferin A, withanolide A, withanoside IV, withanoside V, sominone, and 12-deoxywithastramonolide) [4,5,6,7,8]. The presence of 3*R*-viscosalactone B and the unidentified compound WFNX in urine suggests that our understanding of WS’s metabolism and bioavailability has been limited by withanolide selection in previous studies. A related limitation of our approach, however, is that our detection method was specific to those 11 withanolides and it is likely that additional compounds are present, which could be detected using an untargeted approach.

Another limitation is that we did not verify the efficiency of enzyme hydrolysis using a positive-control enzyme substrate, but relied instead on a protocol that had effectively released phenolic compounds from conjugates in our earlier studies [31,32]. Therefore, while enzyme-catalyzed release of withanolides was observed here for all the analytes, the conditions used may have been suboptimal. The reported amounts of WFNX and 3*R*-viscosalactone B excreted in urine also likely deviate from the true value as they are based on calibration curves prepared for 4-oxo withaferin A and 2,3-didehydrosomnifercin, respectively. We also did not analyze disintegration and dissolution data for the Shoden^®^ capsules, which may have been a factor influencing the amount of withanolides absorbed. Because participants were provided with food two hours after Shoden^®^ administration, it’s possible this affected absorption of withanolides and/or the disintegration and dissolution of the Shoden^®^ capsules.

Another strength of our study was the inclusion of older adults, who are generally underrepresented in clinical trials [33] and are an important population in botanical medicine because age is a predictor of herbal medicine use [34]. Unfortunately, our study is limited by the small sample size (*n* = 4) and specifically, that only one of the four participants was able to complete pharmacokinetics visits for both doses. Our initial recruitment goal was 12 participants, but the study encountered several challenges that impacted the feasibilty of the study design. In addition to intervention-related side effects at the highest dose of Shoden^®^ leading to study withdrawals, some of the challenges encountered (e.g., participants presenting with mild anemia on study days [35], difficulties with venous access) may have been influenced by the age of the study population.

### 3.4. Future Directions

A priority for future investigations is the identification of the metabolite WFNX, which was abundant in all the participant urine samples. The amount of WFNX present in the stored urine aliquots was too small to isolate. In future studies, a larger urine volume could be collected to separate and isolate WFNX. Generation of sufficient quantities of WFNX in vitro using liver microsomal or fecal incubations of withaferin A could also be attempted. The supplier of 4-oxo withaferin A (Cayman Chemicals, Ann Arbor, MI) indicated the compound was synthesized from withaferin A (personal email communication). Isolation and analysis of by-products of the reaction may yield clues as to the identity of WFNX.

Our selection of withanolides to analyze in the urine was based on the availability of reference standards, but given our results, it is likely that other withanolides are also present that we did not detect. An untargeted analysis in urine is a priority as this would provide additional insight into the systemic presence and excretion of both withanolide and non-withanolide components of WS. Finally, comparing the urine data presented here to the plasma data collected from these participants will provide more context for the urine results.

## 4. Materials and Methods

### 4.1. Materials

For method development and preparation of calibration solutions, 11 withanolides were selected as reference standards based on their commercial availability and acceptable purity. Withanolide B [CAS 56973-41-2], 4-oxo withaferin A [CAS 6850-30-2], and 12-deoxywithastramonolide [CAS 60124-17-6] were purchased from Cayman Chemical (Ann Arbor, MI, USA). Sominone [CAS 98569-64-3], withaferin A [CAS 5119-48-2], and 3β-methoxy-2,3-dihydrowithaferin A [CAS 73365-94-3] were purchased from Chemfaces (Wuhan, China). Withanolide A [CAS 32911-62-9] and withanone [CAS 27570-38-3] were purchased from ChromaDex (Los Angeles, CA, USA). Withanoside IV [CAS 362472-81-9] and withanoside V [CAS 256520-90-8] were purchased from TransMIT (Giessen, Hesse, Germany), and 2,3-didehydrosomnifericin [CAS 173614-88-5] was purchased from MedChem Express (Monmouth Junction, NJ, USA). Identity and purity of the reference compounds were confirmed by the Oregon State University Mass Spectrometry Center (OSUMSC) using nuclear magnetic resonance spectroscopy. Compound purity was >95% for most compounds; however, withanolide A (>90%), 3β-methoxy-2,3-dihydrowithaferin A (80%), and sominone (70%) fell below that purity level. Purity was considered in preparing standard solutions. Digoxin-d3 [CAS 127299-95-0], purchased from Cayman Chemical (Ann Arbor, MI, USA), was used as an internal standard.

Pooled human urine was purchased from Innovative research (Novi, MI, USA). For urine sample work-up and LC-MRM-MS, LC-MS grade methanol and acetonitrile (99.9% pure, Honeywell, Burdick and Jackson™) was purchased from Fisher Scientific (Fairlawn, NJ, USA). Solutions (in aqueous glycerol) of sulfatase from *Aerobacter aerogenes* (10–20 units/mL) and β-glucuronidase from *Escherichia coli* (>32,500 units/mL) were purchased from Sigma-Aldrich (St. Louis, MO, USA). Glycerol was obtained from LabChem (Zelienople, PA, USA).

### 4.2. Development of the LC-MRM-MS Method to Measure Withanolides in Human Urine

Method development and analysis of prepared urine samples was conducted at the OHSU Bioanalytical Shared Resource/Pharmacokinetics Core Laboratory (Portland, OR, USA). LC-MRM-MS was conducted using a Sciex 5500 QTRAP hybrid/triple quadrupole linear ion trap mass spectrometer (Redwood City, CA, USA) with electrospray ionization in positive mode. The mass spectrometer was interfaced to a Shimadzu HPLC system (Columbia, MD, USA) with SIL-20AC XR auto-sampler, LC-20AD XR LC pumps and CTO-20AC column oven. The mass spectrometer was operated with the following settings: source voltage 3000 V, GS1 60, GS2 20, CUR 20, TEM 600 and CAD gas medium.

Initial tuning experiments were conducted to identify optimum parent and fragment ions for each withanolide, by infusing individual pure compounds dissolved in a 1:1 mixture of solvent A (water with 0.1% formic acid and 10 mM ammonium formate) and solvent B (acetonitrile with 0.1% formic acid) directly into the mass spectrometer. For 2,3-didehydrosomnifericin, tuning was performed using a solution of the compound in methanol with 10 mM ammonium formate. Chromatographic separation was accomplished using an InfinityLab Poroshell 120 Phenyl-Hexyl (2.1 × 50 mm; 2.7 µm) column with an InfinityLab Poroshell 120 Phenyl-Hexyl (2.1 × 5 mm; 2.7 µm) guard column (Agilent; Santa Clara, CA, USA) held at 31 °C. Gradient elution was performed using mixtures of mobile phase solvent A and solvent B described earlier. Chromatographic conditions were adjusted to ensure that the four isobaric withanolides withaferin A, withanone, 12-deoxywithastramonolide, and withanolide A (RMM 470.6 Da; Figure 1) were well separated and could be distinguished by their retention times. For all subsequent analyses, the following gradient was used: initial concentration of solvent B was 10%, which was held for 0.1 min, followed by a linear gradient to 38% B over 7 min, held at 38% B until 9 min, a linear gradient to 50% B over 1 min, followed by a linear gradient to 98% B over 4 min, then held at 98% B for until 16 min, transitioned back to start conditions (10% B) over 0.2 min, and then re-equilibrated for 3.8 min for a total run time of 20 min. The flow rate was 0.3 mL/min and injection volume was 5 µL.

Withanolides were detected as their molecular ions [M+H]+ or ammonium adducts [M+NH_4_]+ and their fragments using the following MS/MS transitions (m/z): withanone (488.1/263.1; 488.1/165), withaferin A (471.2/281.2; 471.2/175), withanolide A (488.2/263.1; 471.2/399.2), withanolide B (472.3/455.3; 472.3/109.1), 12-deoxywithastramonolide (471.2/263.1; 488.2/471.2), withanoside IV (800.5/459.3; 800.5/423.2), 4-oxo withaferin A (469.2/297.1; 469.2/67), 3β-methoxy-2,3-dihydrowithaferin A (503.3/67.0; 503.3/95), sominone (459.1/67.1; 459.1/287.1), and withanoside V (784.4/443.3; 784.4/425). The withanolide 2,3-didehydrosomnifericin was detected as its molecular ion (489.3/67.0) and its ammonium adduct (506.3/489.3). The bolded transition was used for quantification with an alternate transition used for peak qualification to confirm analyte identity. The internal standard digoxin-d3 was detected as its molecular ion and two fragments (800.5/654.4; 800.5/97.2), both of which were examined for utility in quantitation of the withanolides. A scheduled MRM method was used, with analyte data collected in a 90 s window with 0.2 s dwell time. Peak area data were acquired using Analyst 1.7.1 and processed with Multiquant 3.0.3 software.

To examine the mass spectral fragmentation pathway of 4-oxo withaferin A and the urinary metabolite WFNX, the same LC method was used, but the quadrupole settings were changed such that Q1 captured the ion at m/z 469 and Q3 functioned in enhanced product ion (EPI) mode scanning from m/z 50 to 1000.

### 4.3. Method Validation Experiments

A simple “crash” clean up method of adding organic solvent to urine samples to precipitate salts was adopted. Since participant urine samples would be analyzed with and without prior incubation with glucuronidase and sulfatase enzymes dissolved in 50% aqueous glycerol, this step was included in the validation experiment. Mixtures of the 11 selected withanolides were added to commercial pooled human urine to cover a range of concentrations between 0 and 800 ng/mL. Quintuplicate urine samples (100 µL) at each concentration were mixed with 50% glycerol (sham enzyme, 4 µL) and incubated for 20 min at 37 °C to simulate enzyme digestion. After incubation, a crash solution (400 µL) containing a 4:1 acetonitrile:methanol solution and the internal standard (8 ng/mL digoxin-d3) was added to each urine sample. Samples were held at −20 °C for 30 min to encourage precipitation of salts and then centrifuged at 12,000 rpm (15,280× *g*) for 5 min. Aliquots of the supernatant (450 µL) were then transferred to glass vials and dried using a nitrogen evaporator. Methanol (100 µL) was added to the dried samples and vortexed to ensure mixing. Methanolic solutions were then transferred to centrifugal filters (Nanosep^®^, Pall Laboratory, Port Washington, NY, USA) and centrifuged; the resulting filtrate was transferred to HPLC vials for LC-MRM-MS analysis using the method described earlier (Section 4.2).

Peak areas of each analyte and of the internal standard digoxin-d3 at each of their two respective MRM transitions were obtained using the software described above. For each analyte, the peak area ratio to internal standard (digoxin) was calculated using the analyte’s quantification MRM transition (Section 4.2) and both digoxin MRM transitions. Thus, two sets of peak area ratio vs. concentration values were obtained for each analyte with five replicates at each concentration.

#### 4.3.1. Determination of Selectivity

To confirm that the peak observed for a particular analyte did originate from that compound, detection using both the quantification and qualification transitions (Section 4.2) was confirmed. In addition, samples (100 µL) of pooled human urine without any added withanolides (blank samples) were processed and analyzed as described above, and chromatograms were compared with samples spiked with the withanolides. For participant samples, baseline urine samples obtained prior to administration of Shoden^®^ were compared to pooled urine collected over 12 h following its administration (Section 4.6).

#### 4.3.2. Determination of Precision

For each withanolide, precision of the response was evaluated for each concentration analyzed as the relative standard deviation (RSD) of the 5 replicate peak area ratios corresponding to that concentration. The Food and Drug Administration (FDA) recommends that for bioanalytical methods, an acceptable RSD is ≤15% except at the lower limit of quantitation (LLOQ), where the RSD can be 20% [11].

#### 4.3.3. Determination of Accuracy

A calculated analyte concentration was determined for each standard solution from plots of area ratio vs concentration. Accuracy was determined as the calculated concentration expressed as a percentage of the nominal concentration prepared. FDA guidelines recommend that for bioanalytical methods the accuracy is within 100 ± 15% except at the lower limit of quantitation (LLOQ), where it can be 100 ± 20% [11].

#### 4.3.4. Determination of Range

Precision, accuracy, and linearity were used to determine the working range of the method. The LLOQ was determined as the lowest concentration with RSD ≤ 20% and accuracy of 100 ± 20%. The upper limit of quantitation (ULOQ) was determined as the highest concentration with RSD ≤15%, accuracy within 100 ± 15%, and where there was no discernable curvature in the area ratio vs concentration graph that would indicate possible saturation of the detector signal.

#### 4.3.5. Determination of Linearity

Graphs of area ratio vs concentration were plotted using concentrations within the from 0 to the ULOQ, and their correlation coefficients (R^2^) determined. For the purposes of this study, a correlation coefficient (R^2^) value of ≥0.95 was considered acceptable.

### 4.4. Study Intervention

Shoden^®^ (Arjuna Natural Private Ltd., Kerala, India) is a commercially available, 70% ethanolic extract of *Withania somnifera* root and leaves, standardized to 35% withanolide glycosides and produced in a good manufacturing practice (GMP)-certified facility. Shoden^®^ extract powder and capsules (120 mg and 240 mg extract per capsule with microcrystalline cellulose excipient added to achieve the same total weight) were provided by Arjuna Natural Private Ltd., along with certificates of analysis. Batch numbers for Shoden^®^ powder (SH-PR 106 G/2306/B-11), microcrystalline cellulose (2304061-A), Shoden^®^ 120 mg capsules (SP120-B01/NOV23), and Shoden^®^ 240 mg capsules (SP240-B01/NOV23) were provided by the manufacturer. The content of the 11 selected withanolides in the Shoden^®^ batch used in the capsules was determined at OSUMSC using the LC-MRM-MS method described previously [36], with an additional transition (489.20/67.12) to measure 2,3-didehydrosomnifericin. A previous pharmacokinetic study in 16 males had administered a single-dose of 480 mg Shoden^®^ [4]; for the present study, doses of 240 and 480 mg Shoden^®^ were selected to replicate the findings of the previous pharmacokinetic study, while also generating pharmacokinetic data at a lower dose (240 mg) that had been studied in a clinical trial [37]. The OHSU Research Pharmacy handled the storage and dispensing of Shoden^®^ capsules during the study.

### 4.5. Study Population

Healthy, cognitively unimpaired older adults (≥65 years) were recruited from April 2024 to September 2024. The primary method of identifying potential participants (*n* = 47) was the Research Volunteer Registry, a registry of healthy adults interested in research participation, maintained by the Oregon Clinical and Translational Research Institute (OCTRI) at OHSU. In addition, advertisements were placed in OHSU’s March Wellness newsletter, and the study was added to OHSU’s StudyPages website. Inclusion criteria included a mini-mental state exam score ≥26; normal or clinically not significant 12-lead electrocardiogram; hepatic, renal, and thyroid-stimulating hormone parameters within normal range; hemoglobin and hematocrit levels consistent with FDA recommendations for blood donation; willingness to discontinue all botanical supplements during the study; and willingness to adhere to a special diet during pharmacokinetic study visits. Exclusion criteria included blood donation or participation in drug research study within previous 90 days; allergy to nightshade plants; history of cancer within the last 5 years, except non-metastatic skin cancers; any history of prostate cancer; comorbidities requiring medication such as diabetes, kidney failure, liver failure, hepatitis, blood disorders, hypotension, thyroid disease, respiratory disorders, or cardiovascular disease; symptomatic and untreated urinary tract infection; presence of sleep apnea, moderate to severe restless leg syndrome, major circadian rhythm changes, or narcolepsy; major psychiatric disorder; current smoking, alcohol, or substance abuse; or significant disease of the central nervous system.

### 4.6. Study Procedures

This study was a single-center, randomized, double-blind crossover trial in healthy, older adults. Following enrollment, participants were randomized into one of two sequence groups by the OHSU Research Pharmacy, stratified by sex. Group A received 240 mg Shoden^®^ during Study Period 1 and 480 mg Shoden^®^ during Study Period 2, while Group B received 480 mg Shoden^®^ during Study Period 1 and 240 mg Shoden^®^ during Study Period 2. Each study period began with a 12 h pharmacokinetics visit. Participants were asked to avoid caffeine for the 48 h prior to the visit due to caffeine’s potential effects on drug pharmacokinetics [38]. Participants were also asked to fast the night before the visit. Upon arrival, participants were asked to provide a baseline urine sample. They were then administered an oral dose of Shoden^®^ (240 or 480 mg), and participant urine was collected (and pooled) over the following 12 h for analysis. Participants continued their fast for two hours post-Shoden^®^ administration, after which they were provided with three meals and snacks from a limited menu, provided by OCTRI’s Bionutrition Unit, throughout the rest of the visit.

### 4.7. Urine Sample Preparation and Analysis

Participant urine samples were prepared with and without enzymes and compared against calibration curves prepared with withanolides spiked into blank pooled human urine. Participant urine samples (100 µL), in duplicate, were mixed with an enzyme solution containing *A. aerogenes sulfatase* (2 µL; 0.02–0.04 units) and *E. coli* glucuronidase (2 µL; 65 units) in 50% glycerol and incubated for 20 min at 37 °C (pH not adjusted). A second set of duplicate participant urine samples (100 µL) and calibration samples containing standard withanolides in blank urine (100 µL) were mixed with 50% glycerol (sham enzyme, 4 µL) and incubated for 20 min at 37 °C. After incubation, participant samples and calibration samples were processed further as described under the validation experiment (Section 4.3). In addition, blank calibration samples containing no withanolides were prepared with a crash solution consisting of a 4:1 acetonitrile:methanol solution (400 µL) containing no internal standard to confirm absence of signals interfering with the internal standard. Samples obtained by acid hydrolysis of withaferin A followed by isolation of three hydrolysis products using solid phase extraction and preparative high performance liquid chromatography as previously described [13] were also analyzed. All samples were analyzed using the LC-MRM-MS method described earlier (Section 4.2).

### 4.8. Liquid Chromatography-High Resolution Mass Spectrometry (LC-HRMS) of Selected Withanolide Standards and Participant Urine Samples

LC-HRMS was performed on a Thermo Scientific Orbitrap IQ-X mass spectrometer (Waltham, MA, USA) coupled with a Vanquish UHPLC system (Waltham, MA, USA). Mass spectrometric detection was carried out using electrospray in positive ionization mode with full-scan acquisition over a mass range of m/z 200 to 2000 with high-resolution detection. Mass spectral data were processed using Thermo Scientific Qual Browser software for analysis. Selected processed calibration and participant samples were analyzed by LC-HRMS in this study.

## 5. Conclusions

Despite being one of the most widely used botanical dietary supplements, data on the bioavailability and disposition of WS compounds in humans remains limited. In general, low plasma concentrations have been reported for key withanolides in human pharmacokinetic studies of WS products [4,5,6,7,8,9]. This may be because of poor absorption or availability following oral ingestion, but previous studies have all focused on a limited set of withanolides. Our results suggest that withanolides, specifically withaferin A, may also undergo extensive biotransformation in the body, which would affect the observed plasma and urine concentrations. Therefore, future human pharmacokinetic studies of WS products should analyze additional withanolide metabolites and conduct untargeted analyses to capture the full range of WS compounds present in the human body following oral ingestion of WS.

## Figures and Tables

**Table 1:** Analytical details and validated range for LC-MRM-MS analysis of 11 standard withanolides in human urine.

Analyte	Retention Time (min)	Analyte Transition (m/z)	Digoxin-d3 Transition (m/z)	LLOQ ^a^ (ng/mL)	ULOQ ^b^ (ng/mL)	R^2^ Value ^c^
Withanoside IV	5.72	800.5/459.3	800.5/97.2	≤4	≥800	0.99
			800.5/654.4	≤4	≥800	0.99
2,3-Didehydrosomnifericin	6.39	489.3/67.0	800.5/97.2	9.5	≥95	0.98
			800.5/654.4	≤9.5	≥95	0.99
Sominone	7.42	459.1/67.1	800.5/97.2	12.5–25	≥800	1.00
			800.5/654.4	≤5	≥800	1.00
Withaferin A	7.47	471.2/281.2	800.5/97.2	50–125	≥500	0.99
			800.5/654.4	50–125	≥500	0.99
3β-Methoxy- 2,3-dihydrowithaferin A	7.59	503.3/67.0	800.5/97.2	4–10	60	0.99
			800.5/654.4	4–10	60	0.99
Withanoside V	7.75	784.4/443.3	800.5/97.2	≤4	200	1.00
			800.5/654.4	≤4	200	1.00
12-Deoxywitha- stramonolide	7.90	488.2/471.2	800.5/97.2	≤4	200	0.99
			800.5/654.4	≤4	200	1.00
Withanolide A	8.12	488.2/263.1	800.5/97.2	4–10	≥800	0.99
			800.5/654.4	4–10	≥800	0.99
Withanone	8.29	488.1/263.1	800.5/97.2	4–10	60	0.99
			800.5/654.4	4–10	60	0.99
4-Oxo withaferin A	9.59	469.2/297.1	800.5/97.2	47.4–118.5	≥474	0.99
			800.5/654.4	47.4–118.5	≥474	0.99
Withanolide B	11.13	472.3/455.3	800.5/97.2	≤4	200	0.98
			800.5/654.4	≤4	200	0.98

^a^ LLOQ ranges indicate that the lower value did not meet FDA precision and accuracy criteria, whereas the upper value did; the ≤ symbol indicates the stated concentration met FDA criteria and was the lowest concentration tested. ^b^ ULOQ values with the ≥symbol indicate the stated concentration met FDA precision and accuracy criteria, maintained linearity with lower values, and was the highest concentration tested; a single ULOQ value is the highest concentration tested that met FDA criteria prior to loss of linearity. ^c^ Regression equations are presented in Supplementary Table S1.

**Table 2:** Baseline characteristics of study population.

	Male A	Female A	Female B	Female C
Age, years	72	73	65	75
BMI, kg/m^2^	24.43	22.87	28.71	20.54
MMSE ^a^ score	29	27	28	26
GDS-15 ^b^ score	1	1	0	1

^a^ Mini-Mental State Exam, ^b^ Geriatric Depression Scale-15.

**Table 3:** 12-h urinary excretion of four detected withanolides following oral administration of Shoden^®^ to healthy older adults.

Compound	m/z Transition ^a^	Data Type	240 mg Shoden^®^		480 mg Shoden^®^		
			Male A	Female A	Male A	Female B	Female C
Withaferin A	471.2/281.2	Free ^b^	0.10 µmol	0.41 µmol	0.33 µmol	0.05 µmol	0.65 µmol
		Total ^b^	0.11 µmol	0.41 µmol	0.26 µmol	0.07 µmol	0.79 µmol
		% Conjugated ^c^	9%	0%	0%	29%	18%
		% of Dose ^d^	0.49%	1.84%	0.74%	0.16%	1.78%
3*R*-viscosalactone B ^e^	489.3/67.0	Free ^b^	0.34 µmol	0.73 µmol	1.23 µmol	1.90 µmol	1.11 µmol
		Total ^b^	0.38 µmol	0.63 µmol	1.74 µmol	1.88 µmol	1.21 µmol
		% Conjugated ^c^	11%	0%	29%	0%	8%
		% of Dose ^d^	0.83%	1.59%	1.90%	2.08%	1.32%
WFNX ^f^	469.2/297.1	Free ^b^	0.19 µmol	0.26 µmol	0.42 µmol	0.75 µmol	0.51 µmol
		Total ^b^	0.27 µmol	0.33 µmol	0.41 µmol	1.03 µmol	0.38 µmol
		% Conjugated ^c^	30%	21%	0%	27%	0%
		% of Dose ^d^	N/A	N/A	N/A	N/A	N/A
Sominone	459.1/67.1	Free ^b^	0.00 µmol	0.00 µmol	0.00 µmol	0.00 µmol	0.00 µmol
		Total ^b^	0.09 µmol	0.18 µmol	0.24 µmol	0.15 µmol	0.12 µmol
		% Conjugated ^c^	100%	100%	100%	100%	100%
		% of Dose ^d^	29%	58%	38%	24%	19%

^a^ Internal standard was digoxin-d3 (m/z 800.5/654.4); ^b^ Total and Free analyte were measured in samples treated or not treated, respectively, with glucuronidase and sulfatase enzyme; ^c^ Percentage of total withanolide excreted in conjugated form (released by enzyme treatment), ^d^ Percentage of administered withanolide excreted in free or conjugated form in urine (administered dose is reported in Table 4); ^e^ Quantified as 2,3-didehydrosomnifericin; ^f^—Quantified as 4-oxo withaferin A; N/A—not applicable because WNFX was not detected in Shoden^®^ powder.

**Table 4:** Content of selected withanolides in Shoden^®^ powder.

Withanolide	Mean (±SD) µg/mg Shoden^®^ Powder	Mean µmol/mg Shoden^®^ Powder	Mean µmol per 240 mg Shoden^®^	Mean µmol per 480 mg Shoden^®^
12-Deoxywithastramonolide	0.12 ± 0.01	0.0003	0.06	0.12
2,3-Didehydrosomnifericin ^a^	93.19 ± 3.26	0.19	45.77	91.55
3β-Methoxy-2,3-dihydrowithaferin A	2.58 ± 1.03	0.005	1.23	2.46
4-Oxo withaferin A	0.03 ± 0.01	0.00006	0.02	0.03
Sominone	0.60 ± 0.03	0.001	0.31	0.63
Withaferin A	43.62 ± 4.38	0.09	22.25	44.49
Withanolide A	0.09 ± 0.01	0.0002	0.04	0.09
Withanolide B	<LOD	<LOD	<LOD	<LOD
Withanone	<LOD	<LOD	<LOD	<LOD
Withanoside IV	23.56 ± 1.73	0.03	7.22	14.44
Withanoside V	4.50 ± 0.19	0.006	1.41	2.82

SD = Standard deviation, LOD = Limit of detection, ^a^ Mixture of 2,3-didehydrosomnifericin and 3*R*-viscosalactone B, estimated as 2,3-didehydrosomnifericin.

## Data Availability

The data presented in this study are available on request from the corresponding author. The original data are electronically stored at Oregon Health & Science University.

## Supplementary Materials

The following supporting information can be downloaded at: https://www.mdpi.com/article/10.3390/ijms27125289/s1.

## Abbreviations

The following abbreviations are used in this manuscript:

EPI MS	Enhanced product ion mass spectrometry
GDS-15	Geriatric Depression Scale-15
LC-HRMS	Liquid chromatography coupled to high resolution mass spectrometry
LC-MRM-MS	Liquid chromatography coupled to multiple reaction monitoring mass spectrometry
MMSE	Mini-mental state exam
OHSU	Oregon Health & Science University
OSUMSC	Oregon State University Mass Spectrometry Center
UHPLC	Ultra-high performance liquid chromatography
WS	*Withania somnifera*
