# Supplementary material for: Withanolides Are Detected in Human Urine Following Oral Administration of a Withania somnifera Product
Source: Int J Mol Sci. 2026 Jun 11;27(12):5289. doi: 10.3390/ijms27125289 (PMC13299707; doi:10.3390/ijms27125289)

Supplementary Data

Withanolides are detected in human urine following oral administration of a *Withania somnifera* product

Alex B. Speers [1,2*], Ellala D. Limoico [1,2,3], Axel Lozano-Ortiz [1,2,4], Luke Marney [1,5], Jaewoo Choi [1,5], Sarah A. Barr [6], R. Thomas Williamson [6], Wendy K. Strangman [6], Claudia S. Maier [1,5,7] and Amala Soumyanath [1,2]

[1] BENFRA (Botanicals Enhancing Neurological and Functional Resilience in Aging) Botanical Dietary Supplements Research Center, Oregon Health & Science University (OHSU), Portland, OR 97239, USA
[2] Department of Neurology, Oregon Health & Science University (OHSU), Portland, OR 97239, USA
[3] College of Liberal Arts and Sciences, Portland State University (PSU), Portland, OR 97201, USA
[4] Department of Biology, Portland State University (PSU), Portland, OR 97201, USA
[5] Department of Chemistry, Oregon State University, Corvallis, OR 97331, USA
[6] Department of Chemistry and Biochemistry, University of North Carolina Wilmington, Wilmington, NC 28403, USA
[7] Linus Pauling Institute, Oregon State University, Corvallis, OR 97331, USA
* Correspondence: speers@ohsu.edu

Supplementary Table S1. Regression line equations obtained at selected transitions for area ratio vs concentration curves of 11 withanolides from 0 to the ULOQ (Table 1, main manuscript).

Analyte	Analyte Transition (m/z)	Internal standard (Digoxin-d3) transition (m/z)	Regression line equation Day 1[a]	Regression line equation Day 2	Interday % difference in slope[b]
Withanoside IV	800.5/459.3	800.5/97.2	y = 0.0181x + 0.1157	ND	ND
		800.5/654.4	y = 0.0266x + 0.1836	ND	ND
2,3-Didehydrosomnifericin	489.3/67.0	800.5/97.2	y = 0.0027x + 0.0045	y = 0.0254x + 0.025	162%
		800.5/654.4	y = 0044x + 0.006439	y = 0.0424x + 0.0255	162%
Sominone	459.1/67.1	800.5/97.2	y = 0.0018x + 0.0188	y = 0.007x + 0.0108	118%
		800.5/654.4	y = 0.0027x + 0.0194	y = 0.0117x + 0.0152	125%
Withaferin A	471.2/281.2	800.5/97.2	y = 0.0039x + 0.0571	y = 0.0058x + 0.0102	39%
		800.5/654.4	y = 0.0063x + 0.084	y = 0.0097x + 0.0148	43%
3β-Methoxy-2,3-dihydrowithaferin A	503.3/67.0	800.5/97.2	y = 0.3016x + 0.4915	ND	ND
		800.5/654.4	y = 0.4444x + 0.6907	ND	ND
Withanoside V	784.4/443.3	800.5/97.2	y = 0.0745x - 0.0247	ND	ND
		800.5/654.4	y = 0.1103x - 0.049	ND	ND
12-Deoxywitha-stramonolide	488.2/471.2	800.5/97.2	y = 0.0874x + 0.1867	ND	ND
		800.5/654.4	y = 0.1299x + 0.2875	ND	ND
Withanolide A	488.2/263.1	800.5/97.2	y = 0.0269x + 0.5564	ND	ND
		800.5/654.4	y = 0.0395x + 0.8412	ND	ND
Withanone	488.1/263.1	800.5/97.2	y = 0.2536x + 0.2697	ND	ND
		800.5/654.4	y = 0.369x + 0.5986	ND	ND
4-Oxo withaferin A	469.2/297.2	800.5/97.2	y = 0.0055x - 0.007	y = 0.0016x + 0.0114	110%
		800.5/654.4	y = 0.0087x - 0.0215	y = 0.0027x + 0.0139	105%
Withanolide B	472.2/455.2	800.5/97.2	y = 0.156x + 0.8833	ND	ND
		800.5/654.4	y = 0.231x + 1.2684	ND	ND

a – Range of the graph was from 0 to the upper limit of quantitation (ULOQ) given in Table 1 of the main manuscript. b – Interday % difference in slope calculated as [100 x (larger slope – smaller slope)/ 0.5 x (day 1 slope + day 2 slope)]. ND = not determined.

Supplementary Figure S1. Selectivity of the system for the 4 primary withanolide analytes of interest (withaferin A, 4-oxo withaferin A, sominone and 2,3-didehydrosomnifericin) and the internal standard digoxin-d_3.

For each analyte, chromatograms are shown for the quantifying LC-MRM-MS transition (Supplementary Table 1) for **A.** blank commercial urine, **B**. blank commercial urine spiked with the standard compound, **C**. sample participant baseline urine prior to administration of Shoden®, **D**. sample participant urine collected over 12h following administration of Shoden®, and **E**. (sominone only) sample participant urine collected over 12h following administration of Shoden® and treated with glucuronidase/sulfatase enzyme. For the internal standard (digoxin-d3), chromatograms are shown for the 800.5/654.4 transition that was used for the quantitation (**A.** blank commercial urine, **B**. blank commercial urine spiked with digoxin-d_3).

Withaferin A 471.2/281.2

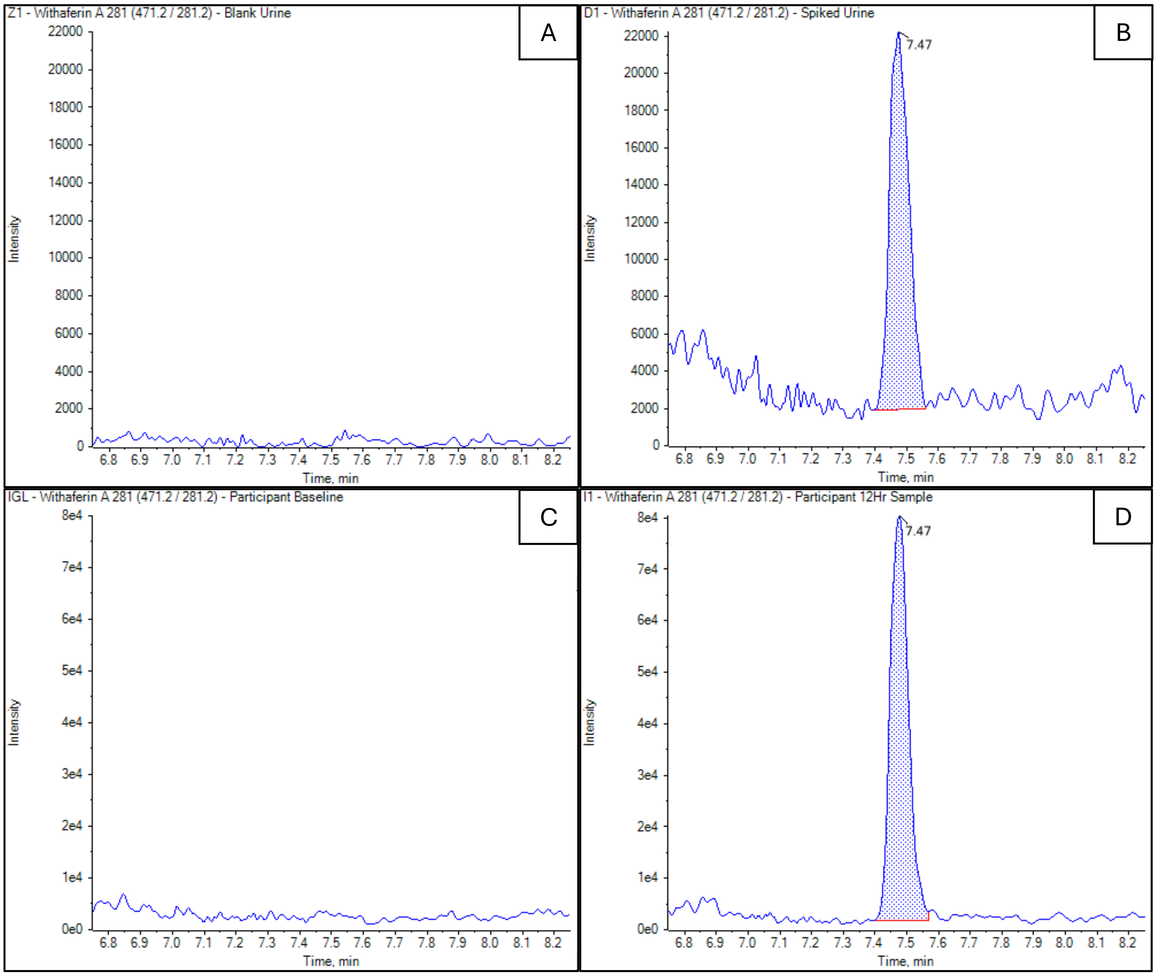

A. blank commercial urine, **B**. blank commercial urine spiked with the standard compound, **C**. sample participant baseline urine prior to administration of Shoden®, **D**. sample participant urine collected over 12h following administration of Shoden®.

4-Oxo withaferin A 469.2/297.1

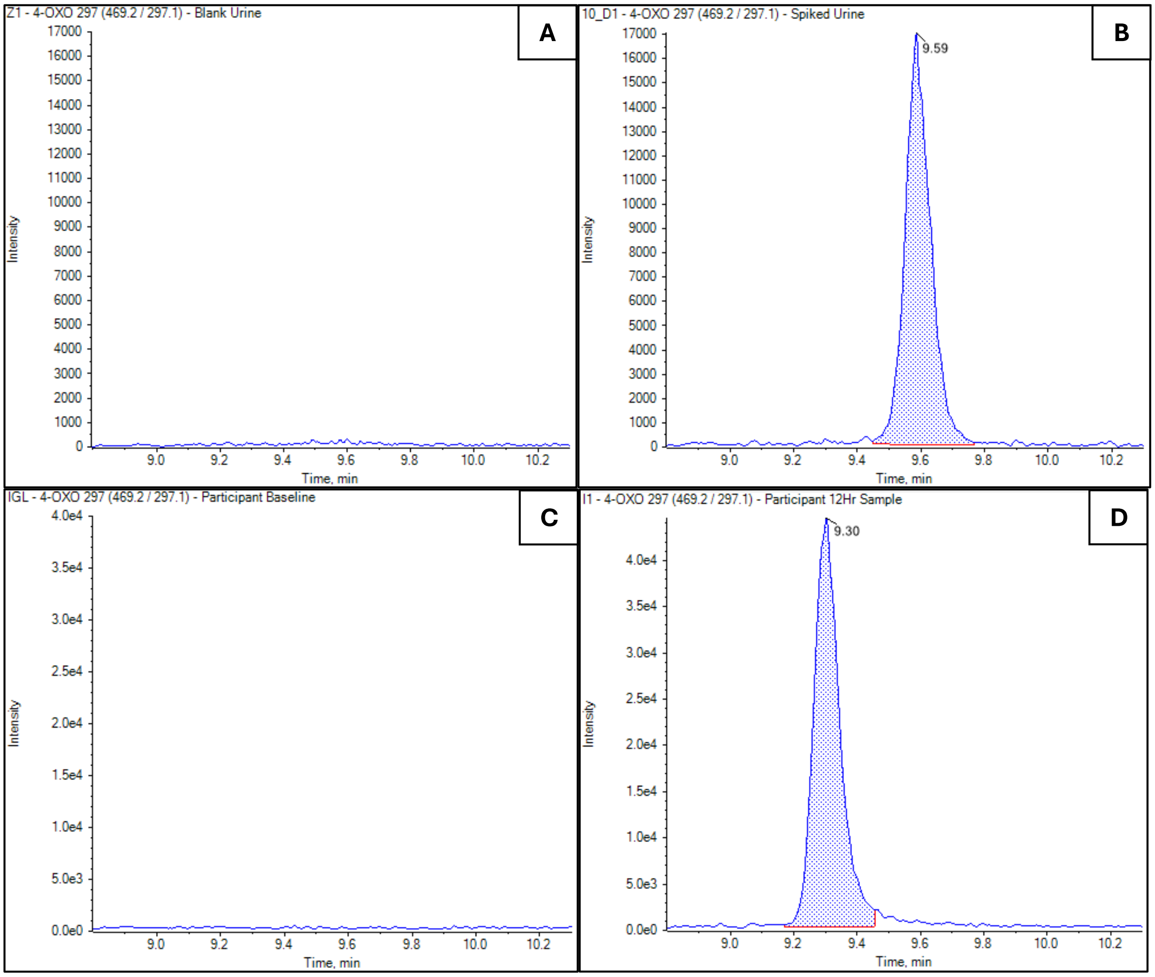

A. blank commercial urine, **B**. blank commercial urine spiked with the standard compound, **C**. sample participant baseline urine prior to administration of Shoden®, **D**. sample participant urine collected over 12h following administration of Shoden®.

Sominone 459.1/67.1

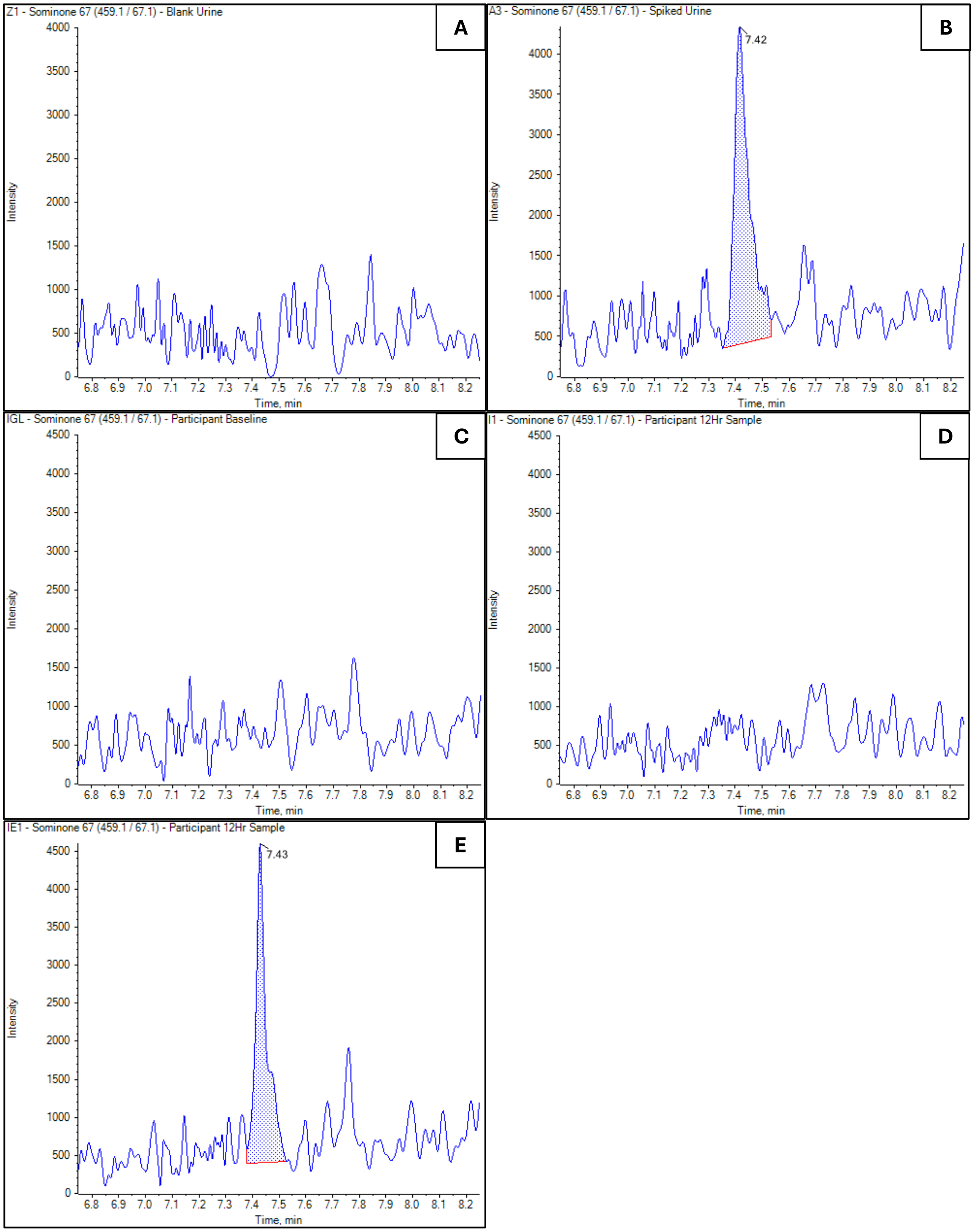

A. blank commercial urine, **B**. blank commercial urine spiked with the standard compound, **C**. sample participant baseline urine prior to administration of Shoden®, **D**. sample participant urine collected over 12h following administration of Shoden®; **E**. sample participant urine collected over 12h following administration of Shoden® and treated with glucuronidase/sulfatase enzyme.

2,3-Didehydrosomnifericin 489.3/67.0

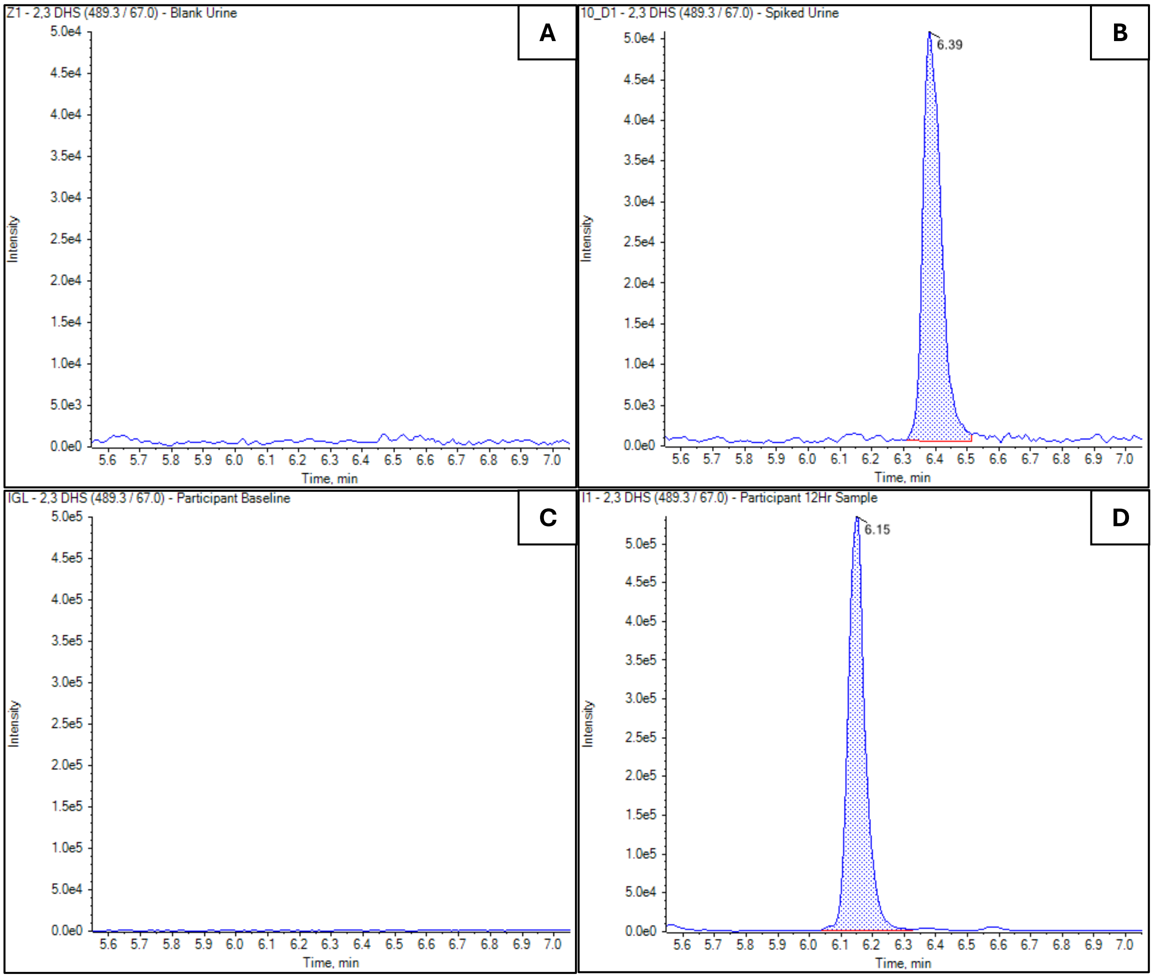

A. blank commercial urine, **B**. blank commercial urine spiked with the standard compound, **C**. sample participant baseline urine prior to administration of Shoden®, **D**. sample participant urine collected over 12h following administration of Shoden®.

Digoxin 801.5/97.2

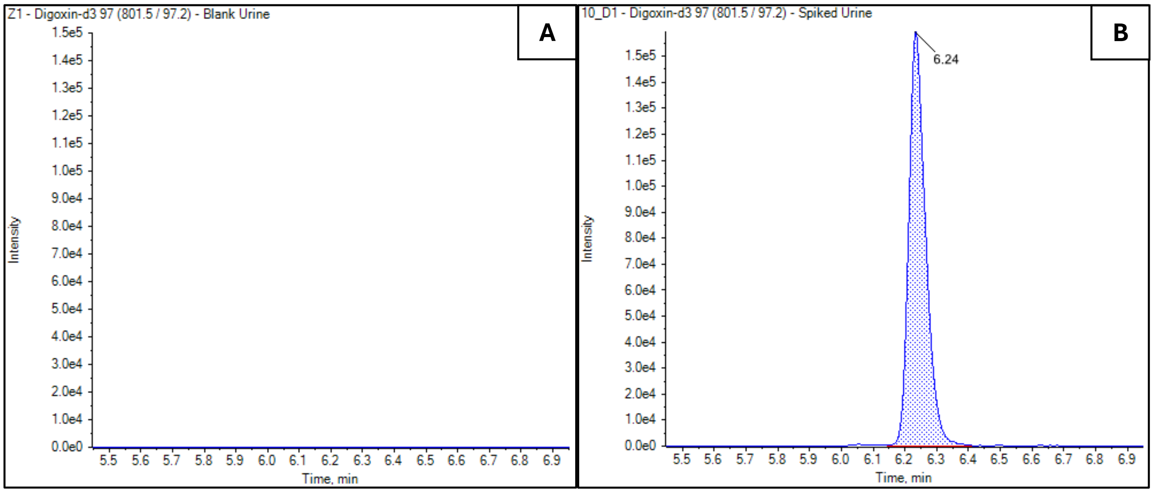

A. blank commercial urine, **B**. blank commercial urine spiked with digoxin-d_3.

Digoxin 801.5/654.4

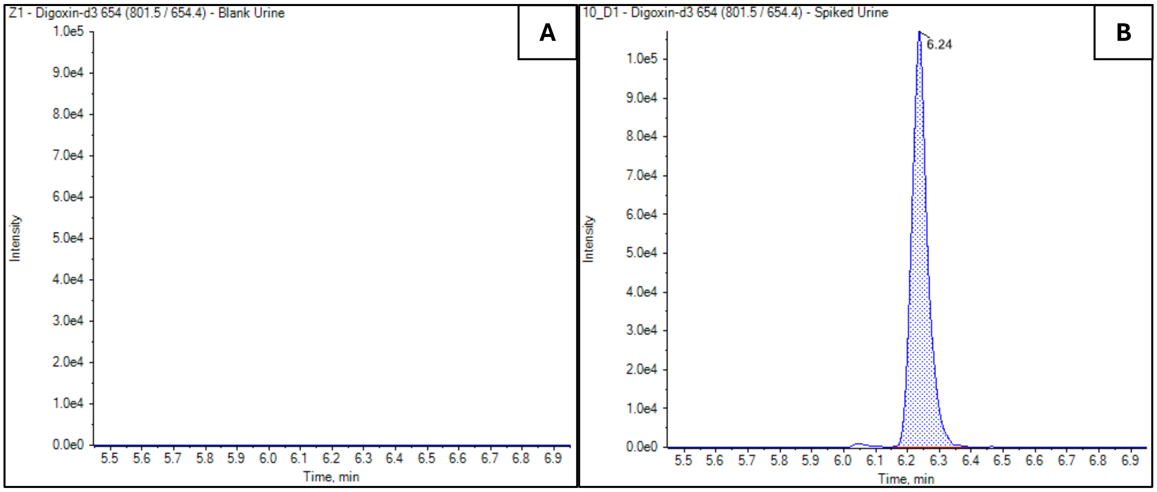

A. blank commercial urine, **B**. blank commercial urine spiked with digoxin-d_3.

Supplementary Figure S2. Liquid chromatography-high resolution mass spectrometry of withanolide standards for 2,3-didehydrosomnifericin and 4-oxo withaferin A and participant urine samples.

Instrumentation: Thermo Orbitrap IQ-X instrument, equipped with a Vanquish Horizon UHPLC system; Scan settings for 4-oxo withaferin A: 469.25797-469.25897, RT 9.2min $C_{28}H_{37}O_6$; Scan settings for 2,3-didehydrosomnifericin: 489.28418-489.28518, RT 6.2min $C_{28}H_{41}O_7$

Standard 2,3-didehydrosomnifericin; m/z 489, RT 6.22min; Mass Error 0.42 PPM

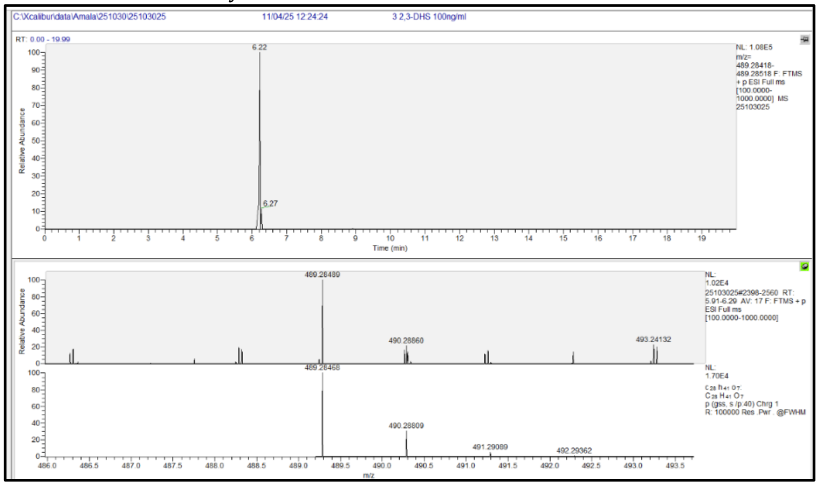

Urine sample, Female B (collected over 12h post Shoden 480 mg); m/z 489, RT 5.91min, Mass Error 0.59 PPM

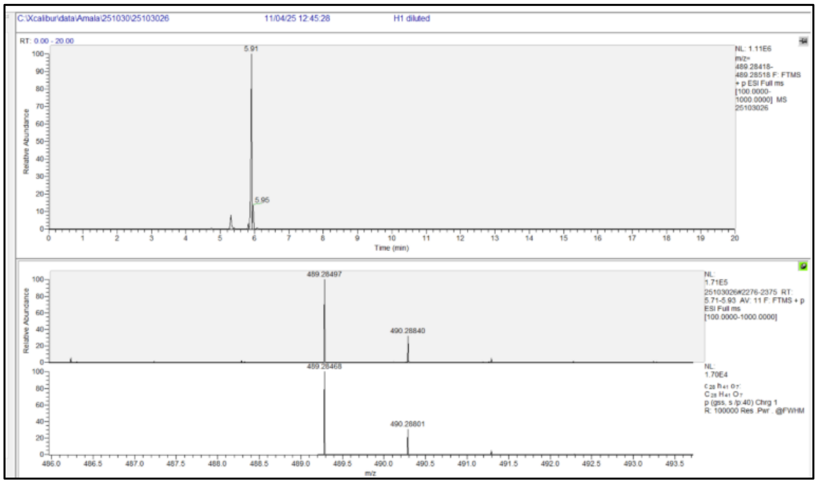

Standard 4-oxo withaferin A (run on 10.30.25); m/z 469, RT 9.27 min, Mass Error 0.49 PPM

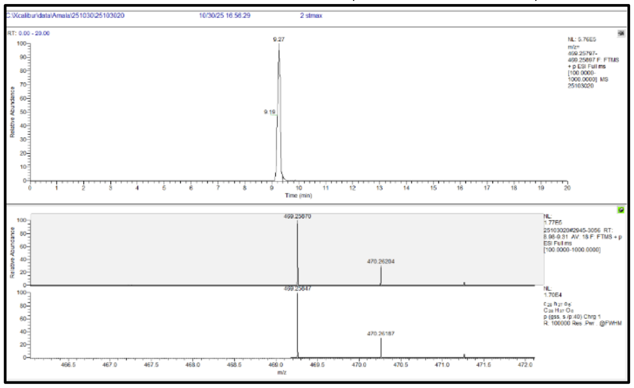

Urine sample, Female B (collected over 12h post Shoden 480 mg); m/z 469, RT 9.1 min, Mass Error 0.51 PPM

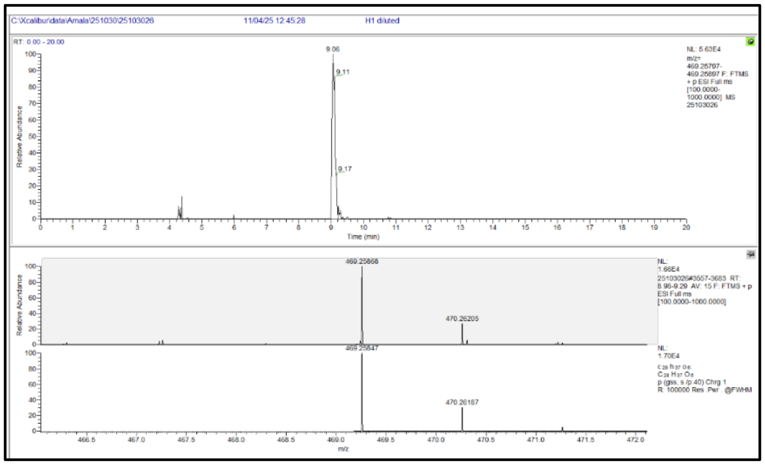

Supplementary Figure S3. LC-MRM-MS analysis of standard 2,3-didehydrosomniferin, isolates from hydrolyzed withaferin A, participant urine and Shoden. Chromatograms obtained using LC-MRM-MS transitions for 2,3-didehydrosomniferin are shown.

Standard 2,3-didehydrosomniferin elutes at 6.31 min (251120 data):

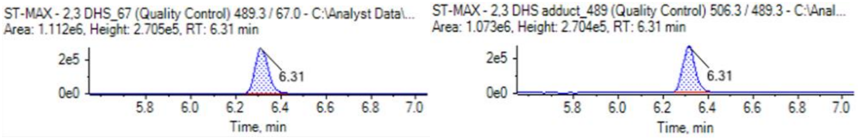

Three peaks purified from the hydrolysate of withaferin A (SAB 1, SAB 3, and SAB 4) elute at 6.06, 6.15 and 6.31 min. SAB-4 corresponded to 2,3-didehydrosomniferin standard (251120 data):

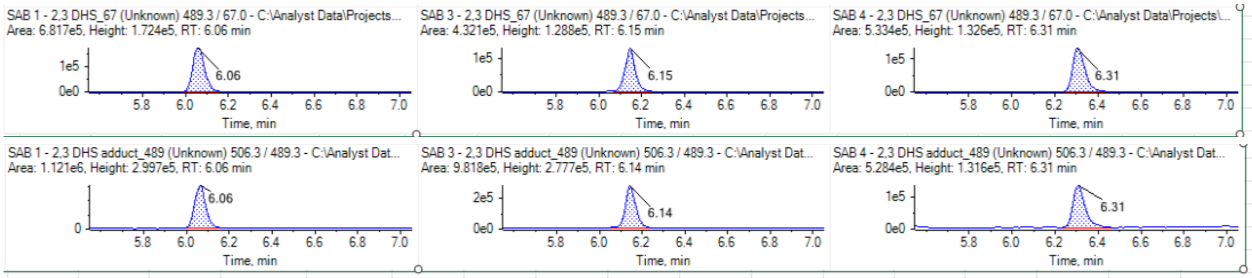

Participant Female B's urine showed a peak corresponding to SAB-3 (260210 data):

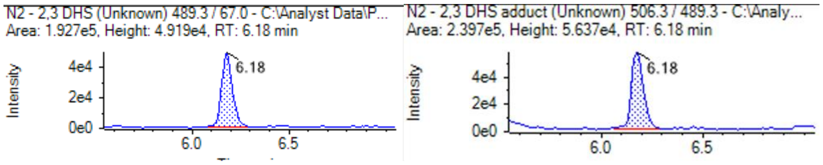

Shoden showed two peaks: the first corresponded to the peak in participant urine and SAB 3, and the second corresponded to 2,3-didehydrosomniferin and SAB-4 (260210 data)

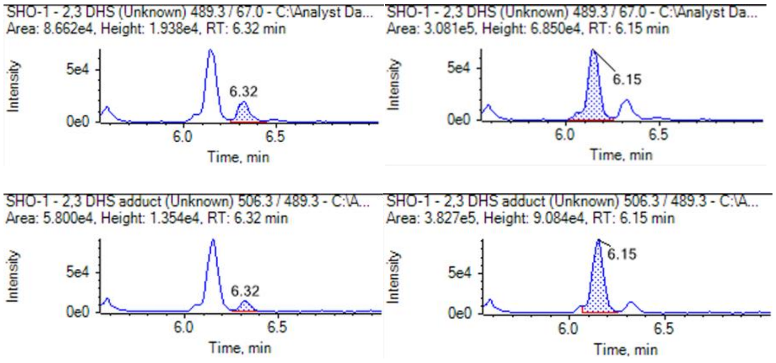

SAB 3 has been identified as 3*R*-viscosalactone B (Barr *et al.*, 2026)

Barr SA, Davis LJ, Vidar W, Williamson RT, Strangman WK. Assessing Digestive Transformations of *Withania somnifera* Extracts via LC-MS/MS Profiling with a Focus on Bioactive Compounds Withaferin A, Withanolide A, Withanoside IV, and Untargeted Metabolomics. J Agric Food Chem. 2026 Mar 18;74(10):8851-8863. doi: 10.1021/acs.jafc.5c09897. Epub 2026 Mar 5. PMID: 41784222.

Supplementary Figure S4. LC-MRM-MS analysis of standard 2,3-didehydrosomniferic in (2,3-DHS) and Shoden® conducted at Oregon State University. Peak detected using the transition 489.2/67.2.

2,3-Didehydrosomniferic in standard (left) vs Shoden® powder (right).
Grey dotted line: predicted Rt 2.54 min; Red dashed line: integration bounds; Red dot and label: measured apex Rt.

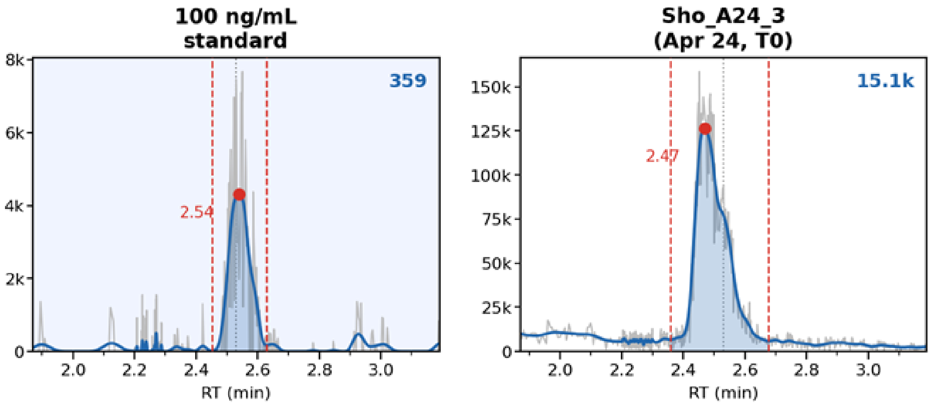

A composite peak was detected in Shoden® for the 2,3-didehydrosomniferic in transition with apex at 2.47 min and a shoulder corresponding in R_t to standard 2,3-didehydrosomniferic in. This agrees with the data shown in Supplementary Figure S3, where these two components of Shoden® are resolved; the larger peak corresponded to SAB 3, identified as 3*R*-viscosalactone B (Barr et al, 2026) and the smaller one to 2,3-didehydrosomniferic in.

Supplementary Figure S5: Comparing Mass Spectra of the standard 4-oxo withaferin A peak and the participant peak using an MS-EPI (enhanced product ion) method with Q1 m/z 469 and Q3 scan from m/z 50-1000

Standard 4-oxo withaferin A peak at Rt 9.38 min; EPI Scan from m/z 50 - 1000

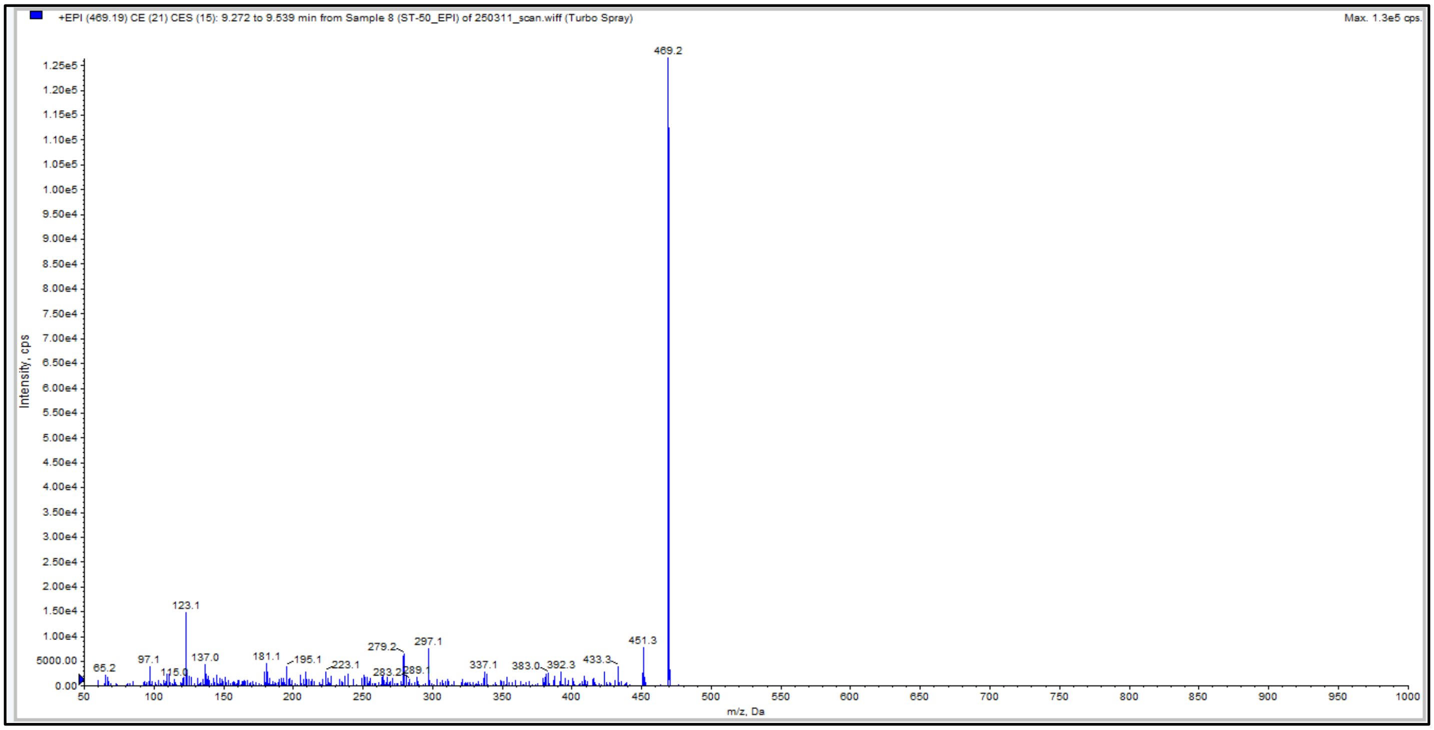

Participant urine WFNX peak at Rt 9.23 min; EPI Scan from m/z 50 – 1000

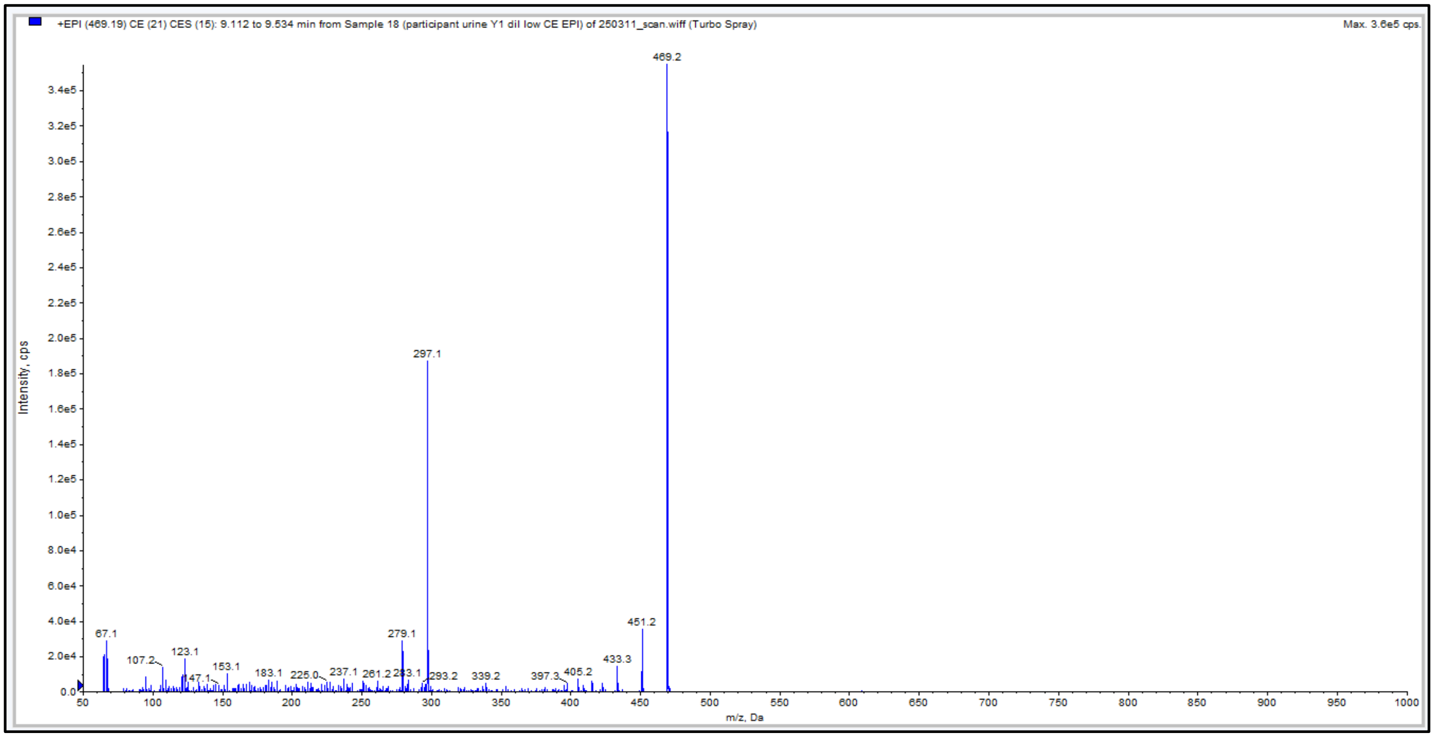

Standard 4-oxo withaferin A peak at Rt 9.38 min; EPI Scan shown from m/z 50 – 460

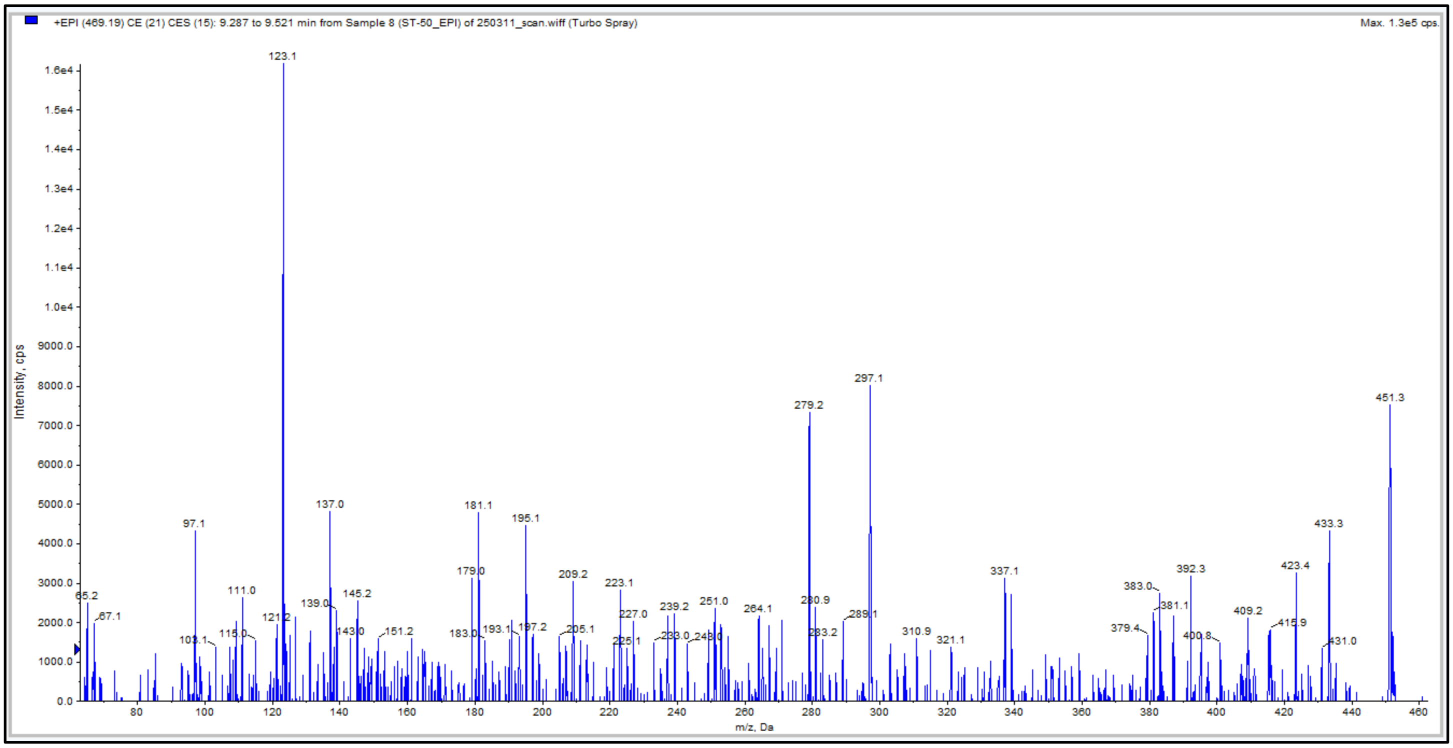

Participant urine WFNX peak at Rt 9.23 min; EPI Scan shown from m/z 50 – 460

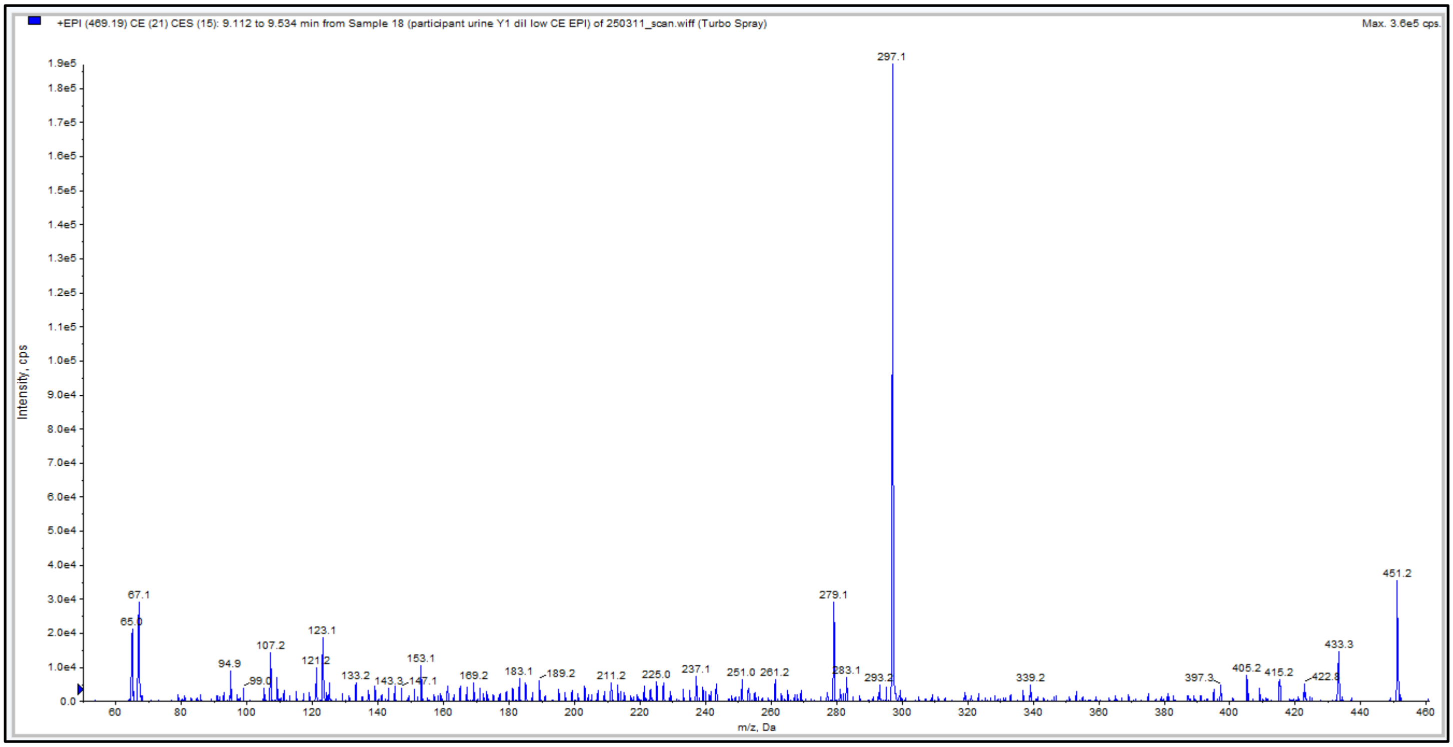

Supplement: Supplementary file 1 [file ijms-27-05289-s001.zip › ijms-4351018-supplementary.pdf]
